# Descriptive data in different paper-based cognitive assessments in elderly from the community Stratification by age and education

**DOI:** 10.1590/1980-57642018dn12-020008

**Published:** 2018

**Authors:** Allan Gustavo Brigola, Ana Carolina Ottaviani, Érica Nestor Souza, Estefani Serafim Rossetti, Mariélli Terassi, Nathalia Alves Oliveira, Bruna Moretti Luchesi, Sofia Cristina Iost Pavarini

**Affiliations:** 1 MSc. Federal University of São Carlos (UFSCar), Graduate Program in Nursing, São Carlos, SP, Brasil.; 2 PhD. Federal University of Mato Grosso do Sul, Três Lagoas, MS, Brazil.; 3 PhD. Federal University of São Carlos (UFSCar), Gerontology Department and Graduate Program in Nursing, São Carlos, SP, Brasil.

**Keywords:** elderly, cognitive assessment, education, primary health care, idosos, avaliação cognitiva, escolaridade, atenção primária à saúde

## Abstract

Cognitive aging is dynamic and heterogeneous in elderly, thus adequate tools such as paper-based tests are relevant to describe the cognitive profile of this population.

**Objective:**

To describe different paper-based cognitive assessments tests in elderly people stratified by age and education.

**Methods:**

A cross-sectional study of 667 elderly (≥60 years) living in the community was conducted. Sociodemographic information was collected. Global cognition was assessed by the Addenbrooke's Cognitive Examination-Revised (ACE-R), Mini Addenbrooke’s Cognitive Examination (M-ACE) and Mini-Mental State Examination (MMSE). The data were analyzed using descriptive statistics, the t-test and Pearson’s Correlation Coefficient.

**Results:**

The findings showed a predominance of women (53.8%), mean age of 71.3 (±7.7) years and 3.6 (±3.5) years of education. The best global cognitive performance and cognitive domain assessment scores were found in the group with higher formal educational level. Each year of education was associated with an increase of up to 10% in scores on the M-ACE and MMSE and up to 11% in ACE-R scores. The mean values of the scores varied according to age, where the 60-69 years group had better scores than other age groups. The correlation matrix between the cognitive tests showed that near perfect correlations (r=1) were frequent in the subgroup with higher education.

**Conclusion:**

Younger elderly and those with higher educational level had greater global and domain scores. This study describes the scores of elderly for different strata of education and age. In practice, it is important to choose the most suitable screening instrument, considering the characteristics of the elderly.

## RESUMO

O envelhecimento cognitivo é dinâmico e heterogêneo, ressaltando a importância de ferramentas adequadas para avaliação da função cognitiva na população idosa brasileira.

**Objetivo:**

Descrever os dados estratificados em idade e escolaridade do desempenho de idosos em diferentes testes cognitivos baseados em papel.

**Métodos:**

Estudo transversal com 667 idosos da comunidade. Informações demográficas foram coletadas. A cognição global foi mensurada pelo Exame Cognitivo de Addenbrooke - Revisado (ACE-R), Mini Exame Cognitivo de Addenbrooke (M-ACE) e pelo Mini Exame do Estado Mental (MEEM). Os dados foram analisados por meio da estatística descritiva, teste T e pelo Coeficiente de Correlação de Person.

**Resultados:**

A maioria dos idosos era mulher (53,8%), as médias foram 71,3 (±7,7) anos para idade e 3,6 (±3,5) anos para escolaridade. O melhor desempenho cognitivo global e nos domínios foram nos idosos com maior nível educacional. Cada ano de escolaridade esteve associado com o melhor desempenho em até 10% nos escores de M-ACE e MEEM e até 11% nos escores de ACE-R. Os valores das médias dos escores variaram entre faixas etárias, sendo que o grupo 60-69 anos apresentou melhor desempenho. A matriz de correlação entre os testes cognitivos mostrou que as correlações próximas à perfeição (r=1) aconteceram frequentemente no subgrupo mais escolarizado.

**Conclusão:**

Idosos mais jovens e com maior escolaridade apresentaram melhores escores cognitivos. Esse estudo oferece uma descrição dos dados dos testes cognitivos considerando faixa etária e escolaridade. Na prática, é consistente que a escolha dos testes para o rastreio cognitivo considere as características da população idosa.

Cognitive performance involves mental functioning, which includes the functions of perception, attention, long-term and short-term memory, logical reasoning, movement coordination, and task planning and execution.^1^ Among the several losses associated with aging, cognitive deficits have the greatest impact on the day-to-day lives of the elderly, their families and the community, owing to the extent of their repercussions and the lack of effective treatments to reverse existing deficits.^2^

Reduced cognitive performance typically occurs during old age, more predominantly in the female population,^3^^,^ 4 among individuals who do not engage in physical activity,^5^^,^ 6 those with a low economic level,^7^ depressive symptoms^8^^,^ 9 and with low functional capacity.^4^

The effects of age and education on performance in neuropsychological tests are consistently observed and represent a crucial variable in the results. These effects have been reported by many studies analyzing the relationship between formal education and low test scores among participants that concomitantly never attended school or have a low educational level^10^^-^ 12 and are elderly.^3^^,^ 10 ^,^ 11

A systematic review of the literature seeking to analyze modifiable risk factors for cognitive performance found that both low educational and socioeconomic level was associated with age-related cognitive decline.^13^ These findings were also evidenced in studies with Brazilian elderly in which a high prevalence of cognitive decline was associated with longevity of the elderly and lower level of education.^4^^,^ 7

An important finding showed that high educational level is considered a protective factor for the development of cognitive disorders, including dementias, and especially for Alzheimer’s disease (AD).^1^ The early detection of cognitive decline in the elderly is an important strategy. This information helps limit the deleterious effects and establish therapeutic plans that can reduce or delay these manifestations.^7^

The high prevalence of cognitive decline is a concern due to the negative impact of cognitive losses on the health and well-being of the elderly. Also, elderly with different age and educational level may have distinct completion times and overall scores on paper-based cognitive tests used. Given the above, the present study aims to describe the data of the cognitive performance scores of elderly living in the community for paper-based neuropsychological tests in an elderly population stratified by age and education.

## METHODS

### Participants

This was a cross-sectional study of elderly residents in the community enrolled at Family Health Units (FHU) of a city in the interior of São Paulo, Brazil. The inclusion criteria were: aged 60 years or over and enrolled at a FHU in the city. This study is a secondary analysis of “The variables associated with cognition in elderly caregivers” study conducted by the Aging and Health Research Group at the Federal University of São Carlos, Brazil.^14^ Exclusion criteria were defined: elderly who had auditory, visual or language limitations precluding completion of the data collection instruments.

The sample was selected from a total of 594 residences listed by the Family Health teams, in which two or more elderly lived; and resulted in 702 elderly interviewed. Of the 702 participants who completed the questionnaire, 37 were excluded from the present analysis because they did not complete the cognitive assessment. The final sample was composed of 667 elderly.

All the ethical care that governs human research has been observed and respected. The project was authorized by the Municipal Health Department of the city and approved by the Research Ethics Committee of the Federal University of São Carlos (Opinion No. 416.467/2013).

### Procedures and measures

Interviews were carried out at domiciles, previously scheduled by trained researchers in Nursing and Gerontology fields. All interviews, lasting approximately one hour thirty minutes, were conducted in a single session between April and November 2014.

Sociodemographic information was collected and included sex (female and male), age (years), education (years), marital status (married, single, divorced and widowed), retirement status (no and yes) and ethnicity (white, brown/mulatto, black).

The assessment of functioning was based on the Katz Index, which analyzes the level of independence for Basic Activities of Daily Living (BADLs), including feeding, continence, transferring, hygiene, dressing, and bathing. The total scale score is calculated as the sum of the items and may range from 0 points (dependent for all functions) to 6 points (independent for all functions).^15^ Instrumental Activities of Daily Living (IADLs) were assessed by the Lawton and Brody Scale, which assesses independence for activities such as performing the housework, handling money, using the telephone, administering medications, traveling, shopping, and preparing full meals. The total score ranges from seven to 21 points, and scores of 7 indicate total dependence, 8-20 points partial dependence, and 21 points independence.^16^

The cognitive assessment entailed application of the Addenbrooke’s Cognitive Examination-Revised (ACE-R), the Mini Addenbrooke’s Cognitive Examination (M-ACE) and the Mini-Mental State Examination (MMSE).

The ACE-R is a battery composed of five domains: orientation/attention, memory, verbal fluency, language and constructive visual ability. The overall battery score ranges from 0 to 100. Cut-off scores were based on educational levels of a Brazilian population living in a community in southeastern Brazil: 0-4 years of education 65 points, and 5 or more years of education 83 points.^10^

The M-ACE was recently devised as a brief test offering similar sensitivity and specificity as its previous versions. The test has cut-off scores for dementia screening with better levels of sensitivity and specificity when compared to the MMSE. The M-ACE is composed of five domains: attention, memory, verbal fluency and constructive visual ability, with a score that ranges from 0 to 30 points, where higher scores indicate preserved cognitive function. For the present study, a cut-off of 21 points was used.^17^

The Mini-Mental State Examination (MMSE) is an instrument that may be used to screen cognitive losses that assesses recent memory and immediate memory recall, temporal and spatial orientation, attention and calculation, and language.^18^ The score ranges from 0 to 30 points. Given the MMSE is a classically used test, several cut-off score can be found in the literature. For the MMSE, three scores described in three different studies were applied: (A) 13 points, 18 points and 26 points (for participants with 0, 1-8 and ≥9 years of education, respectively);^18^ (B) 17 points, 22 points, 24 points and 26 points (for participants with 0, 1-4, 5-8 and ≥9 years of education)^19^ and; (C) 21 points, 22 points, 23 points and 24 points (for participants with 0, 1-5, 6-11 and ≥12 years of education).^20^

### Statistical analysis

The data were analyzed using the Statistical Package for Social Sciences - SPSS software, version 21.0. The descriptive statistics were performed and for the categorical variables a count of simple and percentage frequencies was carried out, whereas for the continuous variables the mean and standard deviation were calculated.

Groups were created according to education (no education=0, 1-4 years of education, 5-8 years and >8 years) and according to age (60-69 years of age, 70-79 years and 80-98 years) and the statistics of the cognitive tests were described by staging (Tables 1 and 2). Additionally, the descriptive data of the instruments for the group with low level of education were calculated, specifically for participants who had between zero and four years of education (Table 3). The independent t-test was performed to compare the scores between education groups. P values ≤0.05 were considered statistically significant on comparisons.

**Table 1:** Mean and standard deviation of cognitive test scores stratified by educational level. Sao Carlos, Brazil, 2014 (n=667).

Education, y	Total (N=667)	0 (n=145)a	1-4 (n=396)b	5-8 (n=62)c	>8 (n=64)d
M-ACE (max 30)	14.6±7.0	8.8±4.0	14.8±6.2	19.4±5.9	21.8±5.9
MMSE (max 30)	21.8±5.3	17.0±4.8	22.4±4.3	25.1±4.2	25.8±5.1
ACE-R (max 100)	58.6±20.8	38.6±14.4	60.0±17.7	73.9±15.3	80.4±16.9
• Attention/Orientation (max 18)	13.8±3.5	10.4±3.4	13.4±2.9	15.1±3.0	15.7±3.2
• Memory (max 26)	13.6±6.4	8.6±4.7	13.7±5.8	18.0±4.8	20.2±5.3
• Verbal Fluency (max 14)	5.2±3.1	2.7±2.3	5.4±2.8	6.9±2.6	8.1±2.8
• Language (max 26)	17.0±6.5	11.0±4.5	17.6±5.8	21.4±4.5	23.0±4.9
• Visuospatial (max 16)	9.4±4.1	5.7±3.2	9.7±3.6	12.3±3.2	13.2±3.2

M-ACE: Mini Addenbrooke Cognitive Examination; MMSE: Mini-Mental State Examination; ACE-R: Addenbrooke Cognitive Examination-Revised. T-test comparative scores with p≤0.05: M-ACE a<b<c=d. MMSE a<b<c=d. ACE-R a<b<c=d. Attention/Orientation a<b<c=d. Memory a<b<c<d. Verbal Fluency a<b<c<d. Language a<b<c=d. Visuospatial a<b<c=d.

**Table 2:** Mean and standard deviation of cognitive test scores stratified by age and educational level. Sao Carlos, Brazil, 2014. (n=667).

Age, y		60-69 (n=328)					70-79 (n=235)					80-98 (n=104)			
Education, y		0 (n=44)a	1-4 (n=199)b	5-8 (n=42)c	>8 (n=43)d		0 (n=54)e	1-4 (n=144)f	5-8 (n=19)g	>8 (n=18)h		0 (n=47)i	1-4 (n=53)j	5-8 (n=1)*	>8 (n=3)*
M-ACE (max 30)		9.3±4.3	16.3±5.8	19.8±6.1	22.9±5.2		9.8±4.7	14.2±6.1	18.5±5.6	20.7±5.6		7.3±4.6	10.8±6.2	22.0	12.3±10.6
MMSE (max 30)		17.8±3.8	23.2±3.5	24.7±4.7	26.4±4.0		17.8±4.4	22.3±4.3	26.0±2.8	25.9±4.2		15.4±5.6	19.6±5.6	25.0	18.0±14.7
ACE-R (max score)		41.3±12.5	64.1±15.5	74.0±16.7	82.9±13.7		40.8±14.7	58.7±17.5	73.2±12.4	78.9±14.5		33.6±14.6	48.3±20.4	83.0	53.6±45.6
• Attention/Orientation (max 18)		1.6±2.6	13.7±2.4	14.8±3.2	15.8±2.9		11.0±3.2	13.5±3.0	15.7±2.2	16.3±2.2		9.4±4.1	12.0±3.6	16.0	11.3±8.9
• Memory (max 26)		9.5±4.8	14.9±5.4	18.3±5.2	21.2±4.7		9.1±4.6	13.5±5.8	17.3±4.0	19.5±4.1		7.1±4.7	9.8±5.4	21.0	9.6±8.3
• Verbal Fluency (max 14)		2.7±2.1	5.9±2.6	7.0±2.7	8.7±2.7		3.0±2.3	5.2±2.9	6.5±2.6	7.1±2.7		2.5±2.3	4.4±3.0	6.0	5.6±4.9
• Language (max 26)		12.5±4.0	18.9±5.1	21.6±4.6	23.7±3.6		11.4±4.3	17.0±5.7	20.8±4.5	22.6±5.0		9.1±4.6	14.0±6.8	25.0	16.3±14.1
• Visuospatial (max 16)		5.8±2.9	10.4±3.3	12.1±3.4	13.3±2.5		6.1±3.8	9.3±3.5	12.7±2.9	13.3±3.3		5.3±2.6	7.9±4.3	15.0	10.6±9.2

M-ACE: Mini Addenbrooke Cognitive Examination; MMSE: Mini Mental State Examination; ACE-R: Addenbrooke Cognitive Examination-Revised. T-test comparative scores with p≤0.05 in 60-69 years old group: M-ACE a<b<c=d. MMSE a<b=c=d. ACE-R a<b<c<d. Attention/Orientation a<b=c=d. Memory a<b=c<d. Verbal Fluency a<b<c=d. Language a<b<c=d. Visuospatial a<b<c=d. T-test comparative scores with p≤0.05 in 70-79 years old group: M-ACE e<f<g<h. MMSE e<f<g=h. ACE-R e<f<g=h. Attention/Orientation e<f<g<h. Memory e<f<g<h. Verbal Fluency e<f=g<h. Language e<f<g=h. Visuospatial e<f<g=h. T-test comparative scores with p≤0.05 in 80-98 years old group: M-ACE i<j. MMSE i<j. ACE-R i<j. Attention/Orientation i<j. Memory i<j. Verbal Fluency i<j. Language i<j. Visuospatial i<j.

* T-test was not performed due the small number of participants in those subgroups

**Table 3:** Mean and standard deviation of cognitive test scores stratified by years of (low) education. Sao Carlos, Brazil, 2014 (n=667).

Education, y	0 (n=145)a	1 (n=48)b	2 (n=65)c	3 (n=74)d	4 (n=209)e
M-ACE (max 30)	8.8±4.0	11.8±5.8	12.0±5.6	14.5±5.9	16.5±3.1
MMSE (max 30)	17.0±4.8	20.0±3.9	20.3±4.7	22.4±4.1	23.7±3.8
ACE-R (max 100)	38.6±14.4	49.5±17.4	51.2±16.8	58.7±16.4	65.7±16.3
• Attention/Orientation (max 18)	10.4±3.4	12.0±2.7	12.2±3.2	13.3±2.8	14.2±2.6
• Memory (max 26)	8.6±4.7	11.0±5.1	11.4±5.3	13.0±5.7	15.4±5.6
• Verbal Fluency (max 14)	2.7±2.3	4.4±2.8	4.2±2.5	5.4±2.8	6.1±2.7
• Language (max 26)	11.0±4.5	14.5±6.7	15.0±5.7	17.3±5.2	19.2±5.3
• Visuospatial (max 16)	5.7±3.2	7.3±3.7	8.3±3.5	9.7±2.8	10.6±3.5

M-ACE: Mini Addenbrooke Cognitive Examination; MMSE: Mini-Mental State Examination; ACE-R: Addenbrooke Cognitive Examination-Revised. T-test comparative scores with p≤0.05: M-ACE a<b=c<d=e. MMSE a<b=c=d=e. ACE-R a<b<c<d<e. Attention/Orientation a<b=c=d=e. Memory a<b=c=d<e. Verbal Fluency a<b=c<d=e. Language a<b=c=d=e. Visuospatial a<b<c<d=e.

Pearson’s Correlation Coefficient was used to analyze correlations between cognitive tests for population and subgroup correlations. Results were considered significant (p<0.01) based on the Bonferroni correction, and the statistic rho ≥9 showed an almost perfect correlation with large effect size (Table 4).

**Table 4:** Pearson Correlation matrix between the cognitive tests, excluding correlation between the specific domains of ACE-R. Sao Carlos, Brazil, 2014.

Assessments	MMSE		M-ACE		ACE-R			MMSE		M-ACE		ACE-R			MMSE		M-ACE		ACE-R	
Statistics	rho	p	rho	p	rho	p		rho	p	rho	p	rho	p		rho	p	rho	P	rho	p
Groups	Total (n=667)							Education: no schooling (n=145)							Education: 1-4 years (n=396)					
M-ACE	.81	<.01						.72	<.01						.76	<.01				
ACE-R	**.90**	**<.01**	**.93**	**<.01**				.87	<.01	.87	<.01				.86	<.01	**.92**	**<.01**		
Attention/Orientation	.94	<.01	.76	<.01	.83	<.01		**.93**	**<.01**	.71	<.01	.81	<.01		**.94**	**<.01**	.68	<.01	.75	<.01
Memory	.78	<.01	**.91**	**<.01**	.89	<.01		.67	<.01	.84	<.01	.84	<.01		.72	<.01	.89	<.01	.86	<.01
Verbal Fluency	.70	<.01	.79	<.01	.81	<.01		.46	<.01	.68	<.01	.59	<.01		.62	<.01	.73	<.01	.76	<.01
Language	.80	<.01	.79	<.01	**.92**	**<.01**		.71	<.01	.58	<.01	.84	<.01		.71	<.01	.72	<.01	.89	<.01
Visuospatial	.75	<.01	.80	<.01	.86	<.01		.56	<.01	.54	<.01	.73	<.01		.69	<.01	.75	<.01	.82	<.01
**Assessments**	**MMSE**		**M-ACE**		**ACE-R**			**MMSE**		**M-ACE**		**ACE-R**			**MMSE**		**M-ACE**		**ACE-R**	
**Statistics**	**rho**	**p**	**rho**	**p**	**rho**	**p**		**rho**	**p**	**rho**	**p**	**rho**	**p**		**rho**	**p**	**rho**	**P**	**rho**	**p**
**Groups**	**Education: 5-8 years (n=62)**							**Education: >8 years (n=64)**							**Age: 60-69 years (n=328)**					
M-ACE	.76	<.01						.86	<.01						.79	<.01				
ACE-R	.89	<.01	**.90**	**<.01**				**.94**	**<.01**	**.92**	**<.01**				.85	<.01	**.93**	**<.01**		
Attention/Orientation	**.95**	**<.01**	.70	<.01	.83	<.01		.95	<.01	.76	<.01	.88	<.01		**.94**	**<.01**	.73	<.01	.81	<.01
Memory	.75	<.01	.86	<.01	.84	<.01		.79	<.01	**.90**	**<.01**	.87	<.01		.76	<.01	**.91**	**<.01**	.89	<.01
Verbal Fluency	.62	<.01	.70	<.01	.74	<.01		.69	<.01	.73	<.01	.77	<.01		.67	<.01	.76	<.01	.80	<.01
Language	.72	<.01	.70	<.01	.89	<.01		.87	<.01	.74	<.01	.81	<.01		.72	<.01	.76	<.01	**.90**	**<.01**
Visuospatial	.64	<.01	.75	<.01	.81	<.01		.72	<.01	.79	<.01	.80	<.01		.72	<.01	.79	<.01	.84	<.01
**Assessments**	**MMSE**		**M-ACE**		**ACE-R**			**MMSE**		**M-ACE**		**ACE-R**								
**Statistics**	**rho**	**p**	**rho**	**p**	**rho**	**p**		**rho**	**p**	**rho**	**p**	**rho**	**p**							
**Groups**	**Age:70-79 years (n=235)**							**Age: 80-98 years (n=104)**												
M-ACE	.80	<.01						.82	<.01											
ACE-R	**.90**	**<.01**	**.92**	**<.01**				**.90**	**<.01**	**.91**	**<.01**									
Attention/Orientation	**.92**	**<.01**	.73	<.01	.81	<.01		**.96**	**<.01**	.81	<.01	.86	<.01							
Memory	.75	<.01	.89	<.01	.87	<.01		.78	<.01	.88	<.01	.87	<.01							
Verbal Fluency	**.97**	**<.01**	.77	<.01	.77	<.01		.70	<.01	.83	<.01	.80	<.01							
Language	.79	<.01	.77	<.01	**.91**	**<.01**		.78	<.01	.72	<.01	**.91**	**<.01**							
Visuospatial	.76	<.01	.78	<.01	.86	<.01		.70	<.01	.75	<.01	.86	<.01							

Statistics in bold: correlations close to perfection (r=1).

## RESULTS

The findings revealed that the sample comprised individuals who were predominantly women (53.8%, n=359), with a mean age of 71.3 (±7.7) years and 3.6 (±3.5) years of education. The participants were mostly married (88.9%, n=593), of white (69.0%, n=460) or brown (20.5%, n=137) ethnicity and retirees (76.8 %, n=512). Regarding functioning, the majority of the elderly 79.6% (n=531) were independent for all BADLs and 73.8% (n=492) had partial dependence for IADLs.

The mean scores on the cognitive tests according to educational level are given in Table 1. For the ACE-R, in the different categories of education, total score was lower than expected (0 to 4 >65 points, 5 or more >83 points). In relation to the domains, the stratum of ≥ 8 was below the cut-off score only for attention/orientation and visuospatial domains. The other education strata scored below the cut-off for most of the domains.

On the MMSE, the education strata remained within the expected cut-off level according to the education categories. Notably, the higher the education level the greater the overall and domain scores on the cognitive tests.

Table 2 shows the values for the cognitive tests stratified by education and age group. The mean scores varied according to age; the 60-69 years age group had higher mean scores across all education strata compared to 70-79 years and 80-98 years age groups. The 80-98 years category had lower scores for all levels of education. On the MMSE, only the 5-8 years category scored within expected cut-offs.

Table 3 presents total and domain scores on the cognitive tests with the sample stratified into ≥4 years of education (considered low education). For each year of education, there was an increase of approximately two points in the mean score on the tests. The stratum of 1 and 2 years of education had similar scores on the cognitive tests. Regarding the total score on the MMSE, only the category of illiterates was below the expected score. For the ACE-R, the total score was not below the cut-off only for the group with 4 years of education.

Table 4 presents the correlation matrix between cognitive tests on which all associations were considered significant p<0.1. Most rho statistics had close to perfect correlations (r≥.9) in groups with high educational level. The only correlation with medium effect (r<.5) occurred in the group with no education. There does not appear to be any tendency as to the groups by age.

**Figure 1:**
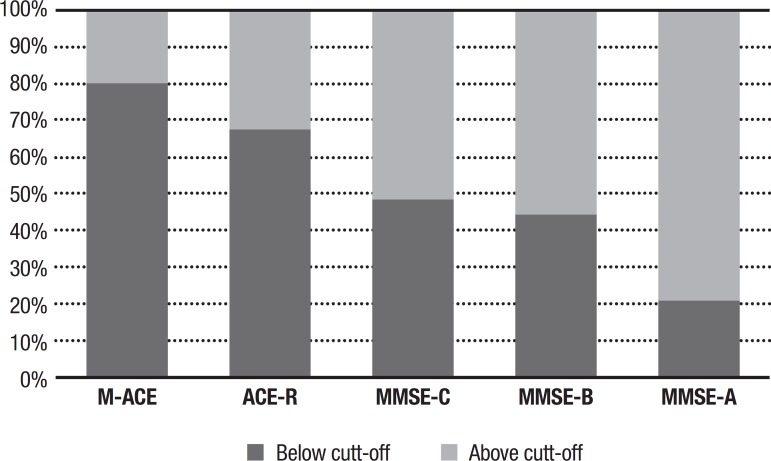
Proportion of participants above and below cut-off scores suggested in studies in the literature. M-ACE: 20.4% above cut-off; ACE-R: 32.4% above cut-off; MMSE-A: 79.2% above cut-off; MMSE-B: 55.6% above cut-off; MMSE-C: 51.4% above cut-off.

Figure 1 shows the proportions of participants who scored above and below the cut-off scores suggested in the literature. The cut-offs B and C suggested for the MMSE showed similar proportions. Both B and C cut-offs agreed for 593 cases and disagreed for 72 cases. The most divergent proportions were for the M-ACE and the MMSE using suggested cut-offs A On this comparison, they agreed for 251 cases and disagreed for 416 cases.

As secondary findings, participants with some dependence and independence for BADLs had statistically different cognitive performances for the MMSE (20.1 versus 22.1, respectively) and ACE-R (59.6 vs 54.5, respectively). For IADL functions, a difference was observed in all the assessments between partially dependent and independent participants. The mean cognitive scores of individuals partially dependent for IADLs (n=492) were similar to the overall sample (Table 1; n=667). In participants independent for IADLs (n=147), the mean scores on each cognitive assessment were up to 10% higher for total scores.

## DISCUSSION

The sociodemographic characteristics of the elderly interviewed proved similar to those observed in Brazilian population studies, with a predominance of participants who were women, married, white, retired and with low educational level.^21^^,^ 22

The initial objective of this study was to describe cognitive performance on cognitive tests for different education and age strata. In general, the higher the level of education, the higher the general and domain scores on the cognitive tests. Education accelerates the processing speed of reasoning, attention, intelligence, executive functions and memory, delaying overall cognitive loss, and improving the performance of the elderly on cognitive tests.^11^ The Shanghai/China survey found that the prevalence of cognitive impairment in elderly who had not completed primary education was 50.5% and that this percentage decreased with increased years of education.^23^

Studies demonstrate that low education is associated with decreased cognitive status as measured by the MMSE, ACE-R and M-ACE-R.^4^^,^ 7 ^,^ 8 ^,^ 10 ^,^ 17 ^,^ 22 ^,^ 24 ^,^ 25 A high educational level is considered a protective factor against the development of dementia conditions, especially AD. The association between low educational level and higher risk of developing dementia conditions may be related to greater exposure to environmental factors, presumably present in individuals with low education.^1^

A systematic review on the cognitive, functional and behavioral assessment of AD found that the MMSE provides good test-retest reliability and diagnostic accuracy, as well as sensitivity and specificity for screening cognitive impairment in dementias.^26^ The ACE-R offers greater sensitivity and predictive value compared to the MMSE for discriminating AD from Frontotemporal Dementia, and is used to detect cognitive impairment.^27^ A recent study that aimed to provide normative data for the total score and domains of the ACE-R in elderly according to educational level, found that total ACE-R scores varied significantly according to age, education and sex.^10^

The present study found that the younger elderly, for different educational levels, had preserved cognitive performance. A higher prevalence of cognitive decline was found in the category of older elderly (≥80 years) and for the lowest level of education, where each year of education was associated with an increase of approximately two points on the mean score of the cognitive tests. Age is considered a predictive factor for cognitive alterations^10^^,^ 11 since the changes resulting from the aging process may lead to complications that compromise different spheres of the individual, such as progressive decline in cognitive functions.^28^

The longitudinal study, based on data from the HEWA study (Health, Well-being and Aging), collected in 2006 and 2010 found that higher education reduced the risk of decline by 62% and 89% for elderly with 4-7 and ≥8 years of education, respectively. In addition, being aged ≤75 years increased the incidence of cognitive decline by 3.29.^22^ The study carried out in the city of Ibicuí in the Northeastern region of Brazil involving elderly with low economic level found a global prevalence of cognitive decline of 18.7%. The highest prevalence of cognitive decline was observed among the older old, aged ≥80 years, women and illiterate individuals. This rate of cognitive decline is alarming given the negative impact of cognitive losses on the health and well-being of the elderly.^7^

Regarding the participants´ cognitive profile in the community, further studies may consider stratifying by other groups, such as those living in rural and urban settings. Some studies found that elderly living in rural environments can have better cognitive performance due less stress and better physical condition.^29^^,^ 30 On the other hand, rural populations can suffer due to distance from health facilities,^31^ whose availability in urban areas tends to be greater.

Based on these results, it can be concluded that higher educational level is directly associated with higher overall and domain scores on all cognitive tests. Each additional year of education influenced final neuropsychological test scores. In addition, younger elderly, with different educational levels, had better cognitive performance on the tests as well as better functional performance. This study reports the scores of the elderly for different education and age strata, with an emphasis on the group with low educational level. The cognitive tests had a great effect in terms of correlations between them, where many displayed almost perfect correlation. In the groups with higher education, the strength of the correlation among the cognitive tests was greater. The proportion of elderly that attained the suggested cut-off scores on the respective tests differed, except for two cut-off scores.

One of the limitations of this study concerns its cross-sectional design, which precluded the identification of the temporal precedence of the cognitive scores and mediating factors. Also, we were unable to provide a clinical evaluation of pre-clinical dementia during this study, which limits some of the interpretations. However, all participants able to answer the questionnaires were included in study, thereby fulfilling the purpose of the study: to describe the cognitive performance of elderly living in the community. These data demonstrate the need for application of screening tools to assess cognitive function of the elderly as part of the routine of health services in primary care to help health professionals provide early preventive and rehabilitation programs. For improved decision-making in care management, it is very important to choose the adequate instrument of cognitive assessment based on the profile of the elderly. Also, the cut-off scores available in literature may represent heterogeneous outcomes, representing a field for future intensive and exhaustive investigation in gerontology in Brazil.
